# Experimental Study of the Effect of Temperature on the Circumferential Bending Performance of GFRP Pipes

**DOI:** 10.3390/polym15020392

**Published:** 2023-01-11

**Authors:** Songdan Luan, Jianzhong Chen, Yong Lv, Xiaoyu Zhang, Li Huang

**Affiliations:** Hubei Key Laboratory of Theory and Application of Advanced Materials Mechanics, School of Science, Wuhan University of Technology, Wuhan 430070, China

**Keywords:** GFRP pipes, temperature, ring stiffness, bending strength, failure modes

## Abstract

Glass Fiber Reinforced Plastic (GFRP) pipes are widely used as polymer-based composite pipes in various engineering fields where the temperature influences their performance. This paper investigated the circumferential bending properties of GFRP pipes with different continuous fiber contents at 30 °C, 50 °C and 70 °C. GFRP pipes are classified into three types according to their component content: type I, type II and type III. The results show that the bending performance of GFRP pipes tends to decrease with increasing temperature, with the retention of circumferential stiffness being 80–85% and the retention of bending strength and damage displacement being about 25–40% from 30 °C to 70 °C. The rate of decay of ring stiffness, bending strength and damage displacement is significantly higher from 30 °C to 50 °C than from 50 °C to 70 °C. Both temperature and continuous fiber content greatly influenced the damage pattern. At 30 °C, delamination damage occurred at the top and bottom of the Type I GFRP pipe before fracture damage happened at the left and right ends and fracture damage occurred at both the left and right ends of Type II and Type III GFRP pipes. Delamination damage happened at the upper and lower ends of the GFRP pipes at 50 °C and 70 °C. In addition, the paper analyses the mechanisms of the associated effects.

## 1. Introduction

Glass fiber reinforced polymer (GFRP) pipes have the advantages of light weight, high strength and corrosion resistance [1,2,3] and are widely used in various industries such as municipal engineering, petrochemical, marine engineering, nuclear engineering, aerospace and automotive [4,5,6,7,8]. A typical GFRP pipe structure consists of three layers: an inner layer, a structural layer and an outer layer. The inner and outer layers are usually made of unsaturated polyester resin, with a structural layer composed of GFRP composites sandwiched between the above two layers [9]. The GFRP pipe used in this paper is made of glass fiber and its products as reinforcing materials, unsaturated polyester resin and other matrix materials and inorganic non-metallic granular materials such as quartz sand and calcium carbonate as fillers, using a continuous winding process method.

Up to now, most studies on the stiffness of GFRP pipes have been conducted mainly under ambient conditions. Zhu et al. [10] experimentally verified the theoretical calculation of the stiffness of a new sandwich-reinforced pipe structure using a flat plate compression test, a finite element model of the sandwich pipe structure was also developed to simulate the compression test and different damage modes were discussed. Chen et al. [11] studied the stiffness of GFRP pipeline under different winding modes and load conditions, established the stiffness degradation model of GFRP pipeline and obtained the parameters of the model. Tu et al. [12] reduced the elastic modulus of GFRP reinforcement by monitoring the strain of GFRP reinforcement in real time and then modeled the degradation of the elastic modulus of GFRP reinforcement in an alkaline, corrosive environment and proposed a relationship between the tensile strength and the elastic modulus of GFRP reinforcement. Rafiee et al. [13] used a simple analytical model based on solid mechanics to estimate the stiffness of the investigated pipes, which in turn predicted the stiffness of GFRP pipes under lateral compressive loads. As research on the stiffness of GFRP pipes at different temperatures is still limited, this paper investigates the effect of temperature on the stiffness of GFRP pipes.

Temperature is an important parameter affecting the mechanical properties of GFRP. Robert et al. [14] investigated the tensile, shear and flexural properties of GFRP steel reinforcement at low temperatures from 0 to 100 °C and at high temperatures from 23 °C to 315 °C, the effects of extreme temperatures on the fibers, matrix and fiber/matrix interface were also investigated and the results showed that the mechanical properties of the composites, especially the stiffness and strength, decrease significantly at very high temperatures close to the glass transition temperature of the polymer matrix. Solyom et al. [15] conducted an experimental and analytical study of the bonding behavior of 8 mm diameter GFRP bars embedded in concrete and exposed to temperatures ranging from −20 °C to 300 °C. Vieira et al. [16] investigated the effect of medium/high-temperature exposure on the residual flexural properties of different types of GFRP commercial pultruded materials. Hosseini et al. [17] initially investigated the tensile strength and modulus of elasticity of four different diameters of GFRP tendons under a four-point bending test at 20 °C. They obtained the tensile strength of different diameters of GFRP tendons at low and high temperatures (−40 to 80 °C) by a four-point flexural test. Lobanov et al. [18] analyzed the degradation of elastic modulus, tensile strength, Poisson’s ratio and strain corresponding to the strength limit with increasing temperature at 22 °C, 100 °C, 175 °C, 250 °C and 300 °C. Liu et al. [19] investigated the bending damage loads, deformation modes and damage mechanisms of carbon fiber-reinforced polymer composite pyramidal truss core sandwich structures in the temperature range of 20 °C to 200 °C; bending stiffness and damage loads were also predicted at different temperatures. Dutta et al. [20] investigated glass fiber-reinforced polyester composites’ tensile and compressive behavior under continuous loading and high temperature. They also developed a simple empirical model using curve-fitting equations to predict failure times. Ashrafi et al. [21] investigated the tensile properties of different GFRP laminates at high temperatures using fiber structure, exposure temperature and laminate thickness as test variables and damage to the resin, fibers and their interfaces was investigated. However, there is still a lack of research on the bending properties of GFRP. Therefore, this paper examines the effect of different temperatures on these bending properties. Yu et al. [22] used different types of epoxy adhesives and FRP bars to prepare NSM FRP specimens and tested the bond strength in the temperature range of 20–400 °C. The experimental results show that the bond strength and modulus decrease significantly in the temperature range of 20~200 °C and only maintain 20~30% of their original values at 200 °C. Correia. et al. [23] studied the tensile, shear and compression responses of GFRP materials at temperatures ranging from 20 °C to 250 °C and analyzed the load-deflection curve, stiffness, failure mode and ultimate strength of GFRP materials. The experimental results show that, due to the glass transition of the resin, the mechanical properties of GFRP deteriorate seriously under moderately high temperatures, especially under shear and compression loads. In Parodi et al. [24], the effect of the thermal history on Tg was investigated by means of fast scanning calorimetry (flash-DSC). The analysis led to the conclusion that the thermal history affects the ratio between rigid and mobile amorphous phases and it is this ratio that determines the glass transition temperature of dry polyamide 6. Alsayed et al. [25] studied the effect of temperature rise on the degradation mechanism of GFRP bars. The results show that the increase in temperature will affect the resin matrix around the glass fiber and thus affect the binding between the fiber and the matrix.

In addition, previous studies have revealed that the fiber volume fraction has an essential effect on the mechanical properties of GFRP pipes. Rafiee et al. [26] investigated the damage process and failure mechanism of GFRP pipes under transverse compressive loading. They found that increasing the core thickness would accelerate in-plane failure and delamination in the pipes. The negative effect of increasing the fiber volume fraction could be compensated by adjusting the winding angle of the cross-ply. In addition, they [27] carried out stochastic modeling of the uncertainty in the discontinuous fiber winding process and found that the effect of changes in fiber volume fraction on the functional failure pressure of GFRP pipes was more pronounced than changes in the winding angle of the spiral plies. Ray et al. [28] investigated the effect of varying the wet thermal conditioning cycle on the moisture gain/loss kinetics and interlaminar shear strength (ILSS) of glass fiber-reinforced epoxy and polyester matrix composites with different mass fractions. However, there is a lack of relevant research on the effect of continuous fiber content on the flexural properties of GFRP pipes. Therefore, this paper further investigates the impact of constant fiber content on the flexural properties by comparing three different types of GFRP pipes.

Most studies have focused on the mechanical properties of glass fiber-reinforced polymers at different temperatures. At the same time, there is a lack of research on the bending properties of different types of GFRP pipes at different temperature conditions. The study of the influence of various factors on these bending properties is of great significance for the structural safety of pipes. Therefore, this paper presents an experimental study of the circumferential bending performance of GFRP pipes with different continuous fiber contents by conducting flat plate external loading experiments at three different temperature conditions of 30 °C, 50 °C and 70 °C. The results of this study will help researchers and design engineers to improve their understanding of the flexural properties of glass fiber-reinforced polymers.

## 2. Materials and Methods

### 2.1. Materials

Glass Fiber Reinforced Plastic (GFRP) pipe is a pipe made of glass fiber and its products as reinforcing material, unsaturated polyester resin and other matrix materials, quartz sand and calcium carbonate and other inorganic non-metallic granular materials as fillers, using fixed-length winding process, centrifugal casting process, or continuous winding process method. The fixed-length winding process is a production method that uses a spiral winding and circular winding process to produce pipes from the inside to the outside of the length of the pipe mold. The centrifugal casting process is a production method in which glass fiber, resin, quartz sand, etc., are cast into a rotating mold by a feeder according to specific requirements and then cured to form a pipe. The continuous winding process is a production method in which resin, continuous fiber, short-cut fiber and quartz sand are continuously layered by the circular winding method according to specific requirements on a constant output mold and cut into a certain length of pipe products after curing.

**Figure 1:**
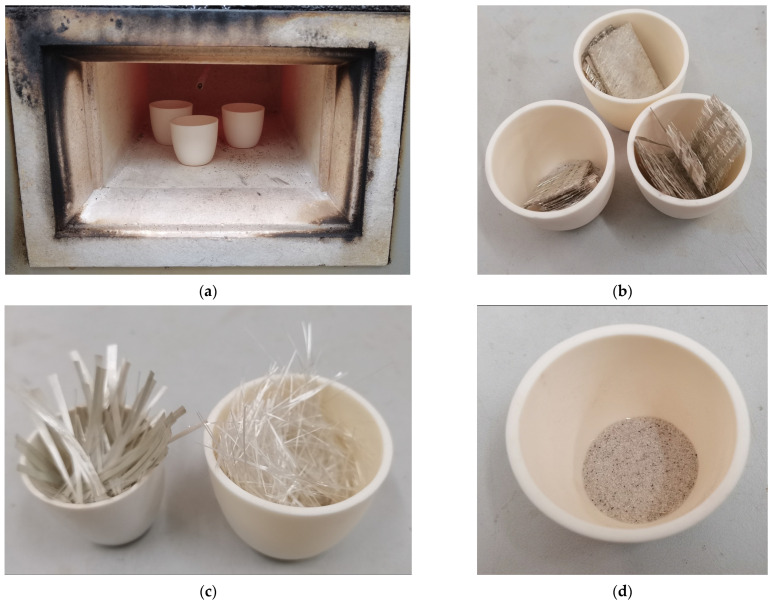
Diagram of the process of measuring the component content of GFRP pipes. (**a**) Thermal decomposition reactions of resins. (**b**) Measuring the content of resin. (**c**) Measuring the content of fiber. (**d**) Measuring the content of quartz sand.

**Figure 2:**
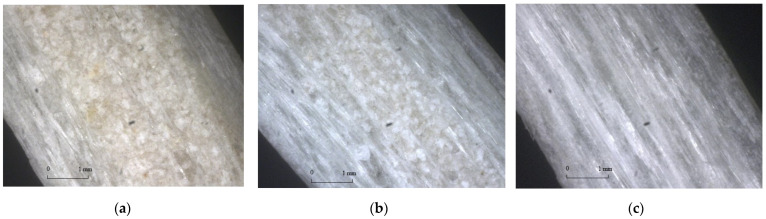
Schematic diagram of GFRP pipe wall structure. (**a**) Type I GFRP pipes (**b**) Type II GFRP pipes (**c**) Type III GFRP pipes.

With the advantages of high equipment productivity, stable product quality and an excellent index of main mechanical properties, continuously wound FRP pipes have become the development trend of the GFRP pipe industry in China. Therefore, the GFRP pipes in this paper are made by a continuous winding process and divided into three types according to the component content. Among them, the component content is measured by SX2-4-10 box-type energy-saving resistance furnace and electronic balance and the specific operation steps are shown in Figure 1. First, the GFRP pipes are cut into small pieces of uniform size and weighed and then put into the box-type energy-saving resistance furnace at 300 °C for thermal decomposition of resin, as shown in Figure 1a, and the component content of the resin is measured when the resin is fully decomposed and the box-type energy-saving resistance furnace is turned off until it drops to room temperature, as shown in Figure 1b. The component content of continuous fiber and short fiber can be measured as shown in Figure 1c; finally, the component content of quartz sand in the beaker is measured as shown in Figure 1d and the specific component content is shown in Table 1. The GFRP tube wall structure was further observed by ultra-deep field 3D microscope VHX-600E and the schematic diagram of the tube wall is shown in Figure 2.

### 2.2. Test Process

To study the effect of different temperatures on the bending performance of GFRP pipes, three groups of tests were designed according to temperatures of 30 °C, 50 °C and 70 °C, with 9 samples in each group, for a total of 27 samples. Among them, three samples of each different type of pipe were tested in each group.

**Figure 3:**
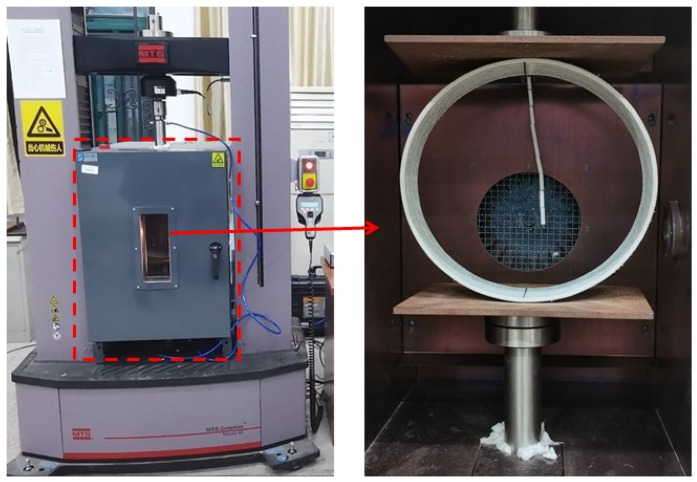
Schematic diagram of determination for external loading properties by parallel-plate loading.

In this test, the MTS C45.105 universal material testing machine was used for the external load test on the flat plate and the GFRP pipe specimen was placed in an environmental chamber equipped with two parallel steel plates. The test equipment is shown in Figure 3. The sample was placed at the center of the loading plate and the initial load was applied to bring the upper loading plate into contact with the sample. The selected test temperatures were 30 °C (room temperature), 50 °C and 70 °C, where the experiment at 30 °C was moved to the condition of room temperature at 30 °C outside of the environmental chamber, due to its size limitation, and the experiments of 50 °C and 70 °C were conducted in the environmental chamber. When the GFRP pipe is heated to the desired temperature in the environmental chamber, it is held for 30 min and then tested to obtain a uniform temperature for the pipe. The flat external load test was performed according to ISO 9969-2016 [29] and loaded at a 6 mm/min loading rate. During the measurements, the loads and displacements were recorded and stored using TWE testing machine software and the damage phenomena were observed by an ultra-deep field 3D microscope VHX-600E.

## 3. Results and Discussion

Ring stiffness of GFRP pipe is an important performance parameter to measure the ability of pipe ring to resist radial deformation under external load. The formula for ring stiffness [30] can be introduced as:

(1) $$SN=0.0186\frac{P}{L\Delta_{Y}}$$

where *SN* is the ring stiffness, N/m^2^; *P* is the radial load applied on the pipe, N; *L* is the length of the pipe, m; $\Delta_{Y}$ is pipe diameter deformation when the radial deformation rate is 3%.

The formula for bending strength [31] can be introduced as:

(2) $$F_{\mathrm{tm}}=\frac{3F_{1}D}{\pi t^{2}}$$

where $F_{\mathrm{tm}}$ is the bending stress, MPa and $F_{1}$, the maximum linear load borne by the pipe ring per unit length along the axial direction, KN/m.

The experimentally obtained data were substituted into Equations (1) and (2) to obtain the corresponding values of ring stiffness and bending strength, as shown in Table 2.

### 3.1. Ring Stiffness

**Figure 4:**
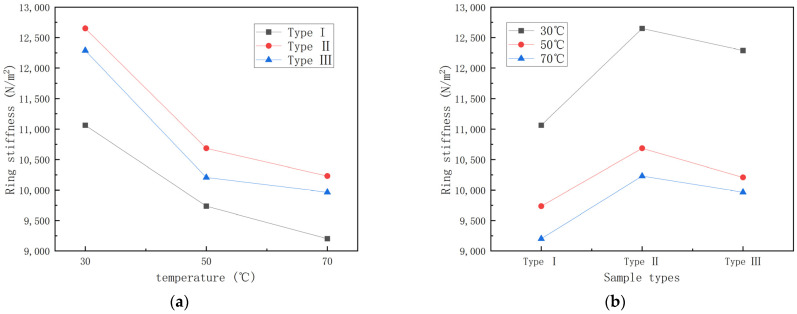
GFRP pipe ring stiffness curve. (**a**) Ring stiffness—temperature curve; (**b**) Ring stiffness—sample type curve.

To investigate the effect of temperature on ring stiffness, the ring stiffness data in Table 2 are plotted as shown in Figure 4 with different temperatures and pipe types as conditions for the ring stiffness-temperature graph and ring stiffness-specimen type graph, respectively.

**Figure 5:**
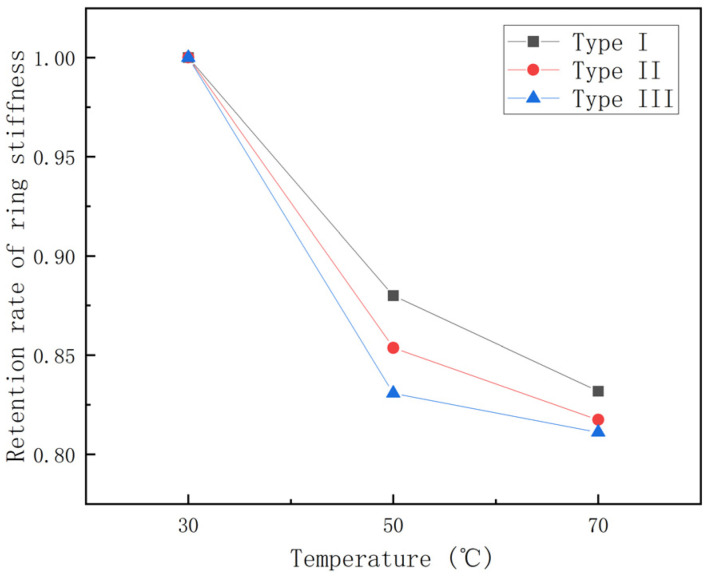
Ring stiffness retention—temperature curve.

**Figure 6:**
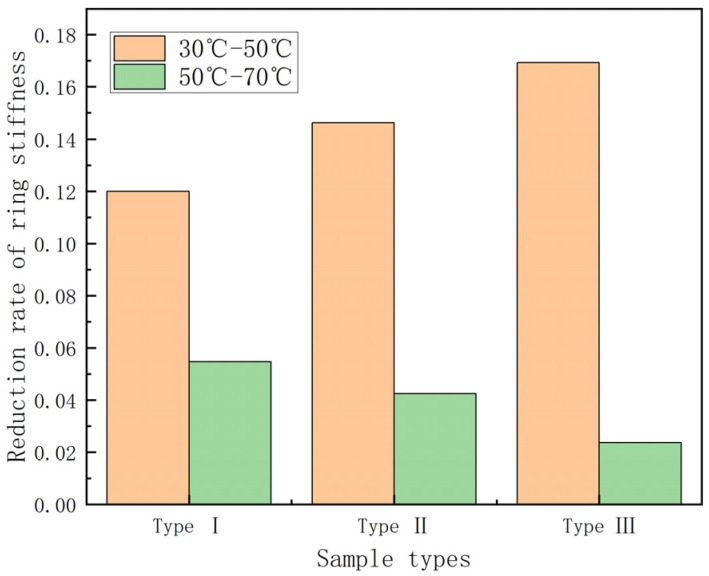
Ring stiffness reduction rate—temperature increment histogram.

From Figure 4, it can be seen that the ring stiffness of different types of GFRP pipes all show a decreasing trend with increase in temperature, among which the ring stiffness of the first type of pipes is the lowest. To further study the effect law of temperature on GFRP pipes, the curves of ring stiffness retention rate for 30 °C rising to 70 °C and the histogram of ring stiffness decay rate per 20 °C rise are plotted, as shown in Figure 5 and Figure 6.

It can be seen from Figure 5 that the retention rate of ring stiffness is 80–85% during the process of temperature rise from 30 °C to 70 °C and the GFRP pipes of the third category are affected by temperature relatively more than the other two kinds of pipes. From Figure 6, it can be seen that the ring stiffness of different types of GFRP pipes decreases faster from 30 °C to 50 °C than that from 50 °C to 70 °C. From 30 °C to 50 °C, the ring stiffness of the pipe decreased by 12–17%, while from 50 °C to 70 °C, the ring stiffness decreased by 2–5%. This is because continuous fiber content plays a significant role in ring stiffness, while temperature has an essential effect on continuous fibers. With the increase in temperature, the elastic modulus of continuous fibers decreases. At the same time, because the strength of the interface between the fiber layer and sandwich layer of GFRP pipes and the fiber and matrix decreases with the increase in temperature, delamination damage is more likely to occur. Therefore, the ring stiffness decreases faster when the temperature rises from 30 °C to 50 °C than when the temperature rises from 50 °C to 70 °C.

### 3.2. Bending Strength

**Figure 7:**
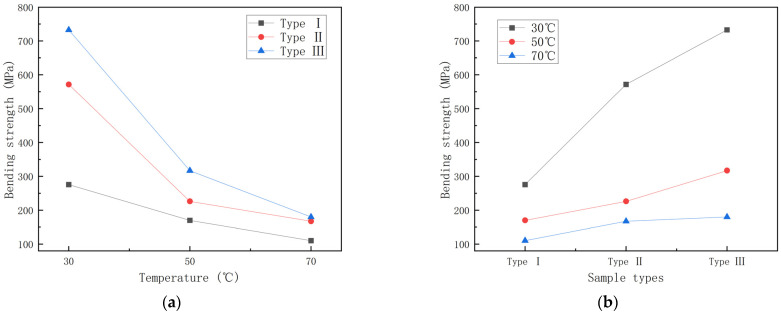
Bending strength curve of GFRP pipes. (**a**) Bending strength-temperature curve; (**b**) Bending strength–sample type curve.

In this section, the effect of temperature on bending strength is investigated. Figure 7 shows the bending strength–temperature graph and the bending strength–specimen type graph for GFRP pipes.

**Figure 8:**
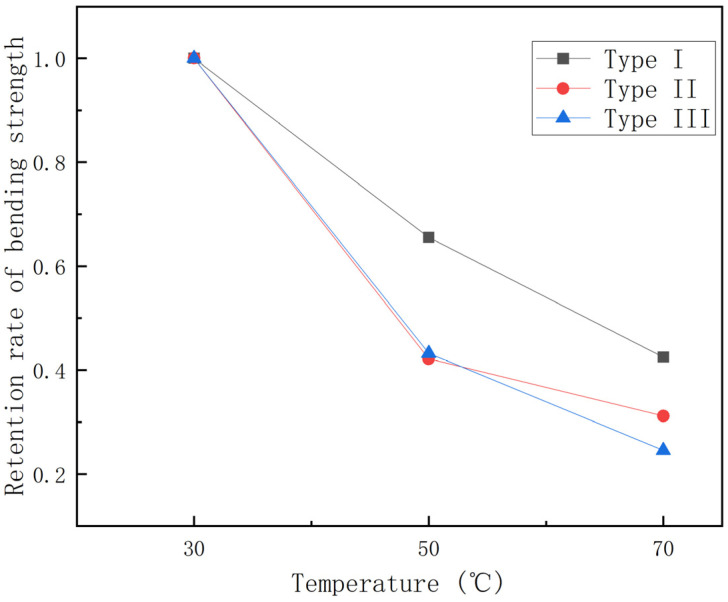
Bending strength retention–temperature curve.

**Figure 9:**
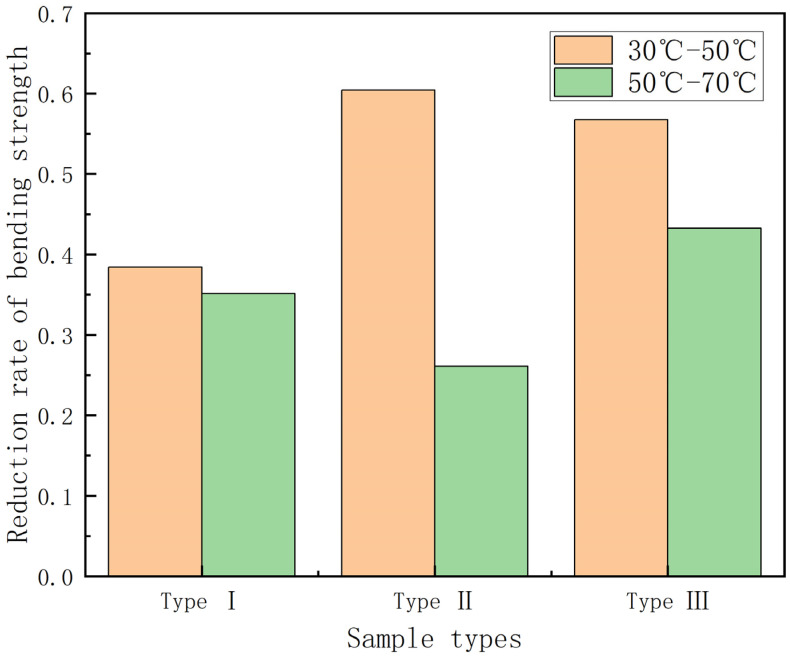
Bending strength reduction rate–temperature increment histogram.

From Figure 7, it can be found that the bending strength gradually decreases with the increase in temperature, with the continuous fiber content gradually increasing, and the bending strength of GFRP pipes of the third category is higher than that of GFRP pipes of the second and first categories at the same temperature. This is due to the fact that continuous fiber content plays a vital role in the bending performance of the composite, so the higher the continuous fiber content, the higher the bending strength of GFRP pipes. To study the effect of temperature on the bending strength more visually, the bending strength retention curve when warming from 30 °C to 70 °C was plotted, as shown in Figure 8, and the histogram of bending strength decay rate per 20 °C warmings, as shown in Figure 9.

As can be seen in Figure 8, the bending strength retention rate is 25–40% during the temperature rise from 30 °C to 70 °C. Among them, the third type of GFRP pipes is more affected by temperature, with a bending strength retention rate of about 25%, while the first type of GFRP pipes has a bending strength retention rate of about 40%. This is because continuous fiber content plays a major role in the bending performance of the composite and the temperature has an important effect on continuous fibers, so the bending strength of GFRP pipes with high continuous fiber content will decrease more quickly than those with low continuous fiber content for the same temperature increment. It can be seen from Figure 9 that the bending strength from 30 °C to 50 °C will decrease more quickly than that from 50 °C to 70 °C. When warming from 30 °C to 50 °C, the effect on the second type of GFRP pipe is very significant, the bending strength decreases by about 60%, while the first type of GFRP pipe bending strength decreases by about 40%. From 50 °C to 70 °C, the third type of GFRP pipe bending strength impact is significant, decreasing by about 40%. This is due to the fact that, when warming from 50 °C to 70 °C, the interface strength between the GFRP pipe fiber layer and sandwich layer and fiber and matrix decreases with the increase of temperature, the damage load decreases, the bending stress decreases and delamination damage occurs more quickly; therefore, the bending strength from 30 °C to 50 °C reduces more quickly compared to that from 50 °C to 70 °C.

### 3.3. Disruption of Displacement

**Figure 10:**
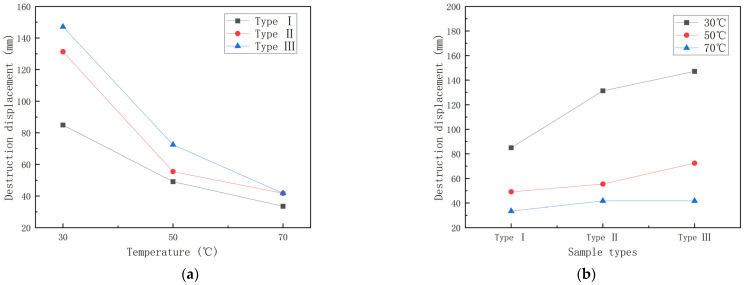
Destruction displacement curve for GFRP pipes. (**a**) Destruction displacement–temperature curve; (**b**) Destruction displacement–sample type curve.

In this section, the effect of temperature on the damage displacement is investigated. Figure 10 shows the graphs of damage displacement–temperature and damage displacement–specimen type for GFRP pipes. In this case, the damage displacement of GFRP pipes with low fiber content uses the displacement value when the damage occurs for the first time.

**Figure 11:**
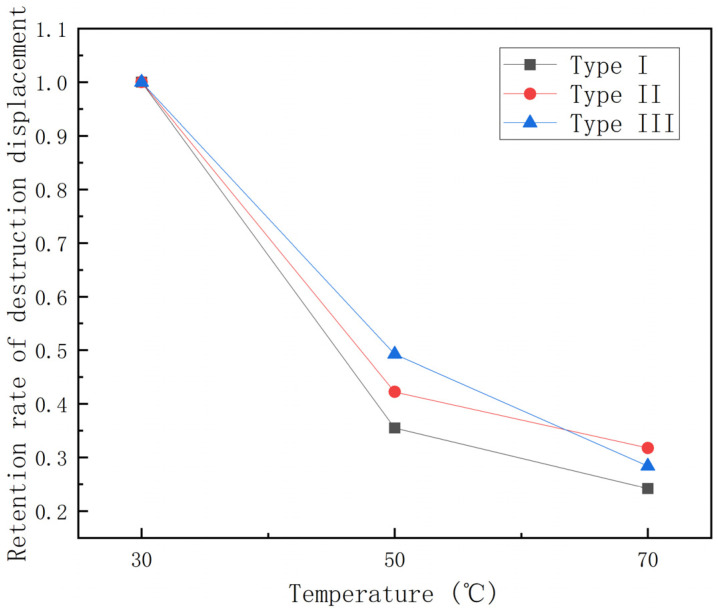
Destruction displacement retention–temperature curve.

**Figure 12:**
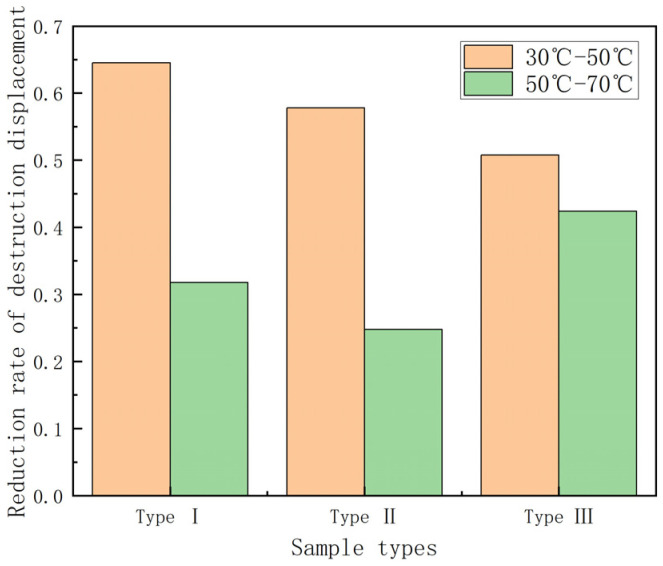
Destruction displacement reduction rate–temperature increment histogram.

It can be seen from Figure 10 that the damage displacement tends to decrease as the temperature increases and, the higher the temperature, the smaller the damage displacement. Under the condition of 70 °C, the effect of temperature on the damage displacement of different kinds of GFRP pipes does not differ much. In addition, under the same temperature condition, the damage displacement gradually increases with the increase of continuous fiber content. Among them, the first type of GFRP pipes underwent damage first. To study the effect of temperature on the damage displacement, the experimental data were plotted as the damage displacement retention rate–temperature graph and the damage displacement decay rate–temperature increment histogram of GFRP pipes, as shown in Figure 11 and Figure 12.

It can be seen from Figure 11 that the destructive displacement retention rate tends to decrease with the increase in temperature and the destructive displacement retention rate is 25%–30% during the process of temperature increase from 30 °C to 70 °C. It can be seen from Figure 12 that the damage displacement from 30 °C to 50 °C decreases quickly compared with that from 50 °C to 70 °C. When warming up from 30 °C to 50 °C, the damage displacement of the first type of GFRP pipes showed a more significant decrease and the damage displacement decreased by about 65%. In contrast, the effect on the third type of GFRP pipes was relatively small and the damage displacement decreased by about 50%. However, when it is warmed up from 50 °C to 70 °C, the impact on the third category of GFRP pipes is more significant and the damage displacement drops by about 40%, while the damage displacement of the first and second category of GFRP pipes drops by 25–30%. This is due to the fact that, when warming from 50 °C to 70 °C, the interfacial strength between the fiber layer and sandwich layer of GFRP pipes and the fiber and matrix decreases with the increase of temperature, the delamination damage is more likely to occur and the damage speed is accelerated. Therefore, the damage displacement from 30 °C to 50 °C decreases faster compared with the damage displacement from 50 °C to 70 °C.

### 3.4. Failure Mode

**Figure 13:**
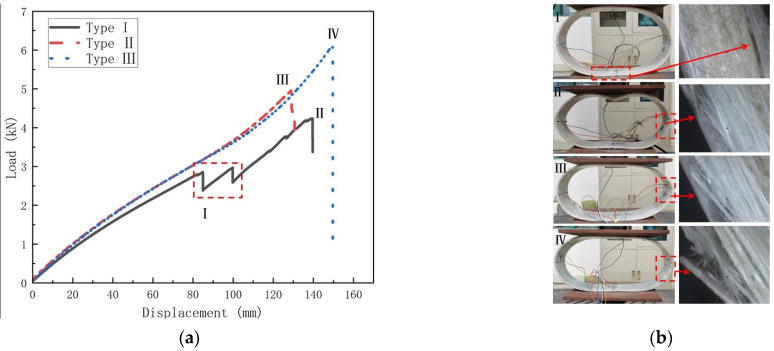
Load–displacement curves and failure mode diagrams for different types of GFRP pipes at 30 °C (**a**) Load–displacement curve for GFRP pipes; (**b**) Failure mode.

**Figure 14:**
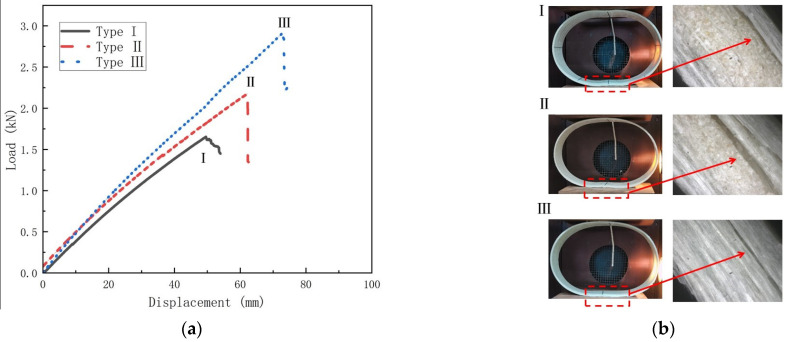
Load–displacement curves and failure mode diagrams for different types of GFRP pipes at 50 °C (**a**) Load–displacement curve for GFRP pipes; (**b**) Failure mode.

**Figure 15:**
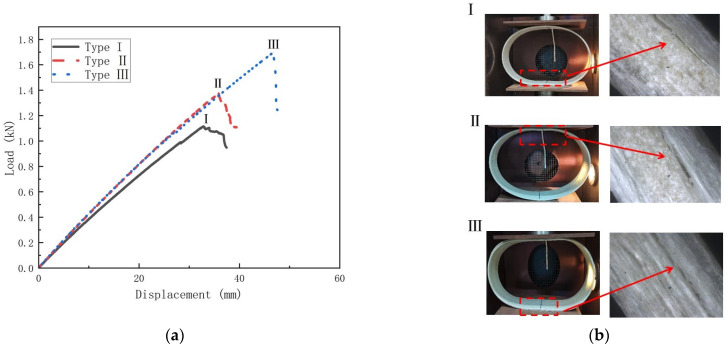
Load–displacement curves and failure mode diagrams for different types of GFRP pipes at 70 °C (**a**) Load–displacement curve for GFRP pipes at; (**b**) Failure mode.

This section investigates the damage modes and damage mechanisms of temperature on GFRP pipes. Figure 13, Figure 14 and Figure 15 show the load–displacement curves and damage modes of GFRP pipes at 30 °C, 50 °C and 70 °C, respectively. Among them, the experiments at 50 °C and 70 °C were conducted only until the first damage due to the size limitation of the equipment.

As can be seen from Figure 13, the damage mode of the first type of GFRP pipe can be divided into two stages under the condition of 30 °C. In the first stage, with gradual increase of the load, the deformation of the pipe increases and, when it reaches point I, the interlayer separation damage occurs between the sandwich layer and the fiber winding layer at the upper and lower ends of the specimen, and the damage mode is shown in Figure 13b. This is because between the sand layer and the fiber layer is the position of the weak force, the upper and lower end of the pipeline bear the maximum bending moment and the inside and outside of the pipeline are in the opposite state of tension and pressure; with the increase in load, the upper and lower ends of the pipeline stress increase, the interlayer shear stress also increases and cracks appear between the sand layer and the fiber layer until complete stratification. In the second stage, as the load continues to increase, the deformation of the pipe continues to increase and, when it reaches point II, fiber fracture damage occurs at the left and right ends of the specimen and the damage pattern is shown in Figure 13b, at which time the specimen is completely damaged and cannot continue to carry the load. This is because the strain at the left and right ends is one half of the strain value at the upper and lower ends. As the load continues to increase, the total deformation increases and the left and right ends of the pipeline first reach the bending strength limit, resulting in fiber fracture. The load of the second and third types of GFRP pipes increases with the increase of displacement. When the ultimate load is reached, fiber fracture damage occurs at the left and right ends of the specimen with a crisp fracture sound, which is due to the gradual increase of the strain of the pipe along with the gradual increase of the load. The left and right ends of the pipe reach the bending strength limit, damage occurs and the damage mode is shown in Figure 13b III and IV.

From Figure 14 and Figure 15, it can be seen that the load-displacement curve trends of different types of GFRP pipes are approximately the same at 50 °C and 70 °C. The damage pattern is delamination at the pipes’ upper and lower ends, which is due to the weakening of the interfacial strength between the matrix and the fibers with the increase in temperature. Thus delamination damage is more likely to occur. However, based on the trend of the damage load–displacement curve and the experimental phenomenon, it can be assumed that the GFRP pipes at 50 °C and 70 °C can continue to be loaded until the fiber fracture damage occurs at the left and right ends of the pipes.

## 4. Conclusions

In this paper, the effect of temperature on the circumferential bending performance of GFRP pipes was investigated and the circumferential bending performance of GFRP pipes with different continuous fiber contents was experimentally studied under the three different temperature conditions of 30 °C, 50 °C and 70 °C. The following conclusions were drawn.

With the increase in temperature, the ring stiffness, bending strength and damage displacement of GFRP pipes show a decreasing trend. The retention rate of ring stiffness is 80–85%, the bending strength retention rate is 25–40% and the damage displacement retention rate is 25–30% during temperature rise from 30 °C to 70 °C.

By comparing the two warming processes, the decay rates of ring stiffness, bending strength and damage displacement during the warming process from 30 °C to 50 °C are significantly higher than those during the warming process from 50 °C to 70 °C.

Both temperature and continuous fiber content have essential effects on the damage mode. At 30 °C, the first type of GFRP pipe undergoes upper and lower end delamination damage followed by left and right end fiber fracture damage, while the second and third types of GFRP pipes undergo left and right end fiber fracture damage. At 50 °C and 70 °C, the upper and lower end delamination damage occurred in GFRP pipes.

By considering different temperatures and different types of GFRP pipes, the effect of temperature on the circumferential bending performance of GFRP pipes was studied and the mechanism of the impact of temperature on ring stiffness, bending strength, damage displacement and failure mode was analyzed. The results of this study provide a solid support for the study of temperature on the bending performance of GFRP pipes.

## Figures and Tables

**Table 1:** Component content data for GFRP pipes.

Sample Types	Resin	Continuous Fibers	Short-Cut Fibers	Quartz Sand
Type I	0.36	0.18	0.12	0.34
Type II	0.37	0.32	0.10	0.21
Type III	0.32	0.51	0.17	-

**Table 2:** Experimental data results of GFRP pipes.

Sample Type	Temperature (°C)	Destruction Load (KN)	Destruction Displacement (mm)	Bending Strength (MPa)	Ring Stiffness (N/m²)
Type I	30	2.65	138.28	275.68	11,063.83
	50	1.67	49.09	169.75	9736.27
	70	1.15	33.50	110.11	9202.93
Type II	30	5.21	131.31	571.43	12,650.00
	50	1.89	55.44	226.11	10,684.91
	70	1.59	41.72	167.10	10,230.50
Type III	30	5.81	147.11	732.48	12,287.10
	50	2.52	72.44	316.88	10,207.32
	70	1.49	41.74	179.83	9965.52

## Data Availability

Not applicable.
